# Cellular microarrays for assessing single-cell phenotypic changes in vascular cell populations

**DOI:** 10.1007/s10544-023-00651-5

**Published:** 2023-03-16

**Authors:** E. Smith, M. Zagnoni, M. E. Sandison

**Affiliations:** 1 Electronic & Electrical Engineering, Royal College Building, University of Strathclyde, G1 1XW Glasgow, UK; 2 Biomedical Engineering, Wolfson Centre, University of Strathclyde, G4 0NW Glasgow, UK

**Keywords:** Single cell, Microwell array, Smooth muscle, Phenotypic modulation, oxLDL

## Abstract

Microengineering technologies provide bespoke tools for single-cell studies, including microarray approaches. There are many challenges when culturing adherent single cells in confined geometries for extended periods, including the ability of migratory cells to overcome confining cell-repellent surfaces with time. Following studies suggesting clonal expansion of only a few vascular smooth muscle cells (vSMCs) contributes to plaque formation, the investigation of vSMCs at the single-cell level is central to furthering our understanding of atherosclerosis. Herein, we present a medium throughput cellular microarray, for the tracking of single, freshly-isolated vSMCs as they undergo phenotypic modulation *in vitro*. Our solution facilitates long-term cell confinement (> 3 weeks) utilising novel application of surface functionalisation methods to define individual culture microwells. We demonstrate successful tracking of hundreds of native vSMCs isolated from rat aortic and carotid artery tissue, monitoring their proliferative capacity and uptake of oxidised low-density lipoprotein (oxLDL) by live-cell microscopy. After 7 days *in vitro*, the majority of viable SMCs remained as single non-proliferating cells (51% aorta, 78% carotid). However, a sub-population of vSMCs demonstrated high proliferative capacity (≥ 10 progeny; 18% aorta, 5% carotid), in line with reports that a limited number of medial SMCs selectively expand to populate atherosclerotic lesions. Furthermore, we show that, when exposed to oxLDL, proliferative cells uptake higher levels of lipoproteins, whilst also expressing greater levels of galectin-3. Our microwell array approach enables long-term characterisation of multiple phenotypic characteristics and the identification of new cellular sub-populations in migratory, proliferative adherent cell types.

**Graphical abstract:**

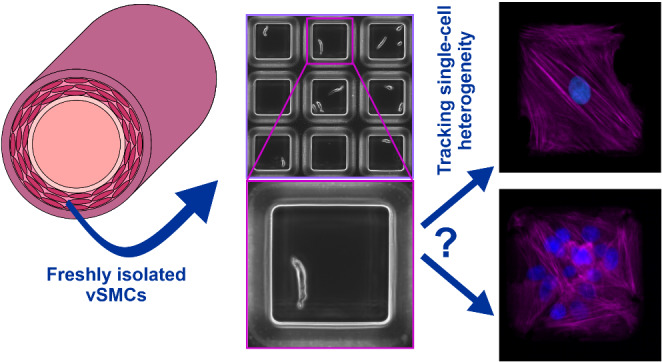

**Supplementary Information:**

The online version contains supplementary material available at 10.1007/s10544-023-00651-5.

## Introduction

Smooth muscle cells (SMCs) play a central role in the remodelling of the vascular wall that underlies atherosclerosis. Vascular SMCs (vSMCs) are highly plastic cells, with the ability to radically alter their phenotype in response to environmental changes (Gomez et al. 2013; Feil et al. 2014; Shankman et al. 2015; Chappell et al. 2016). Fully differentiated vSMCs can rapidly transition from a mature, contractile phenotype to a migratory, phagocytic phenotype (Sandison et al. 2016), coinciding with the down regulation of mature, contractile SMC specific markers (Rong et al. 2003; Chakraborty et al. 2021; Chou et al. 2021) and an increase in capacity for proliferation (Bennett et al. 2016; Yu et al. 2015). However, it is now well accepted that there is substantial heterogeneity within vSMC populations and that vSMCs can display a broad spectrum of phenotypes (Allahverdian et al. 2018). The development of advanced in vivo lineage tracing methods during the last decade has enabled several new discoveries. Notably, this includes a greater understanding of the extent of vSMC contribution to neointima and plaque formation and the previously unrecognised contribution of vSMCs to foam cell formation (Herring et al. 2014; Shankman et al. 2015; Wang et al. 2019). Furthermore, multicolour reporter system models have enabled detailed *in vivo* clonality studies that have suggested clonal expansion of only a few medial SMCs contributes to plaque formation (Chappell et al. 2016; Jacobsen et al. 2017).

The existence of substantial cell-to-cell variability, further evidenced by recent single-cell transcriptomics studies (Dobnikar et al. 2018; Wirka et al. 2019), implies that the use of collective bulk analysis techniques may miss rare but crucial vSMC sub-populations and behaviours. Complementary to *in vivo* studies, the development of new single-cell *in vitro *techniques could offer a route to detailed investigation of cell heterogeneity, combining the ability to perform multiplexed, temporal studies with increased throughput. Previous reports on tracking the fate of single vSMCs *in vitro *using standard culture systems (Sandison et al. 2016) relied on extensive time-lapse microscopy, which is often low data-throughput and time-consuming in nature. Although automated microscopy and high content imaging equipment can be used to substantially improve the throughput of time-lapse data acquisition (e.g. automatically moving the stage to sequentially image across multiple regions), such systems are costly, not routinely available and the tracking of highly proliferative, migratory cells and their progeny can remain challenging and time-consuming. However, novel microengineering technologies can facilitate these studies, as they are ideally suited to the creation of miniaturised single-cell culture tools compatible with standard microscopes.

A wide array of microengineered systems have been developed for single-cell analysis, including microdroplet, microtrap and microarray approaches (Frisk et al. 2011; Yeh and Hsu 2019; Hyman et al. 2021; Yellen et al. 2021; Yao et al. 2019), offering bespoke solutions for large throughput studies and enabling precise control over the manipulation and spatial separation of cells. Many of these systems have been developed for handling cells in suspension or for immediate cell analysis (rather than maintenance *in vitro*), or employ microwell array based systems for the 3D culture of multicellular aggregates (e.g. spheroids and organoids, for example Mulholland et al., 2018, Kakni et al., 2020). Several devices have, however, been tailored for the separation, containment and identification of individual adherent cells. These have employed a range of different architectures and materials, including hydrogel based systems (Roccio et al., 2012), SU-8 micropallets (Wang et al. 2008) and mostly commonly poly(dimethylsiloxane) (PDMS) based devices (Huang et al. 2023; Rettig and Folch 2005; Park et al. 2015; Oyama et al. 2018; Ochsner et al. 2007; Han et al. 2021). Such microwell array based systems have been developed for a range of applications, including single-cell proliferation and apoptosis assays for screening drug responsiveness, and can achieve high single-cell occupancy across arrays and straightforward compatibility with microscopy-based analysis. However, the majority of reported microwell systems have focussed on short-term culture (e.g. a few hours to a few days), with only a few reports containing results from culture periods beyond 3 days (Wang et al. 2008; Cordey et al. 2008). Challenges remain over maintaining the confinement of proliferative and migratory adherent single-cells when cultured for prolonged periods in microwells. Cells can overcome restrictive cell-repellent boundaries with time, for example as a consequence of serum protein adsorption (Li et al. 2015), enabling them to migrate and proliferate outside their assigned confining wells. This impacts negatively on reliable cell tracking and data extraction when monitoring large numbers of single-cells.

Here, we report on the development of a new methodology using a microwell array device for long-term tracking of single vSMC fate. To the best of our knowledge, this is the first time such an approach to single vSMC phenotypic characterisation has been taken. To ensure robust confinement of these highly adherent and migratory cells – which we demonstrate cannot be confined by native PDMS alone and thus approaches relying on PDMS hydrophobicity cannot be successfully employed – we investigated different surface functionalisation approaches, obtaining durable containment (> 3 weeks) of vSMCs and their progeny using the compound Lipidure®-CM. We have employed this system to track the fate of single, freshly isolated vSMCs from two vascular beds (aorta and carotid artery) as they are induced to undergo phenotypic modulation *in vitro*. A combination of live-cell and fixed-cell imaging was used to quantify their proliferative capacity, uptake of oxidised low density lipoprotein (oxLDL) and expression of Galectin-3 (encoded by the gene LGALS3), a commonly used marker of a phagocytic, macrophage-like phenotype highly expressed in atherosclerotic plaques (Alencar et al. 2020). Whilst the majority of confined cells remained as single, undividing cells, a small sub-population of vSMCs were highly proliferative, with the uptake of oxLDL by proliferative cells being significantly greater than that of non-proliferative cells. Our results demonstrate that this microwell array approach is highly amenable to the study of dynamic phenotypic diversity within adherent cell populations, allowing detailed characterisation of multiple phenotypic characteristics and the identification of new cellular sub-populations.

## Materials and methods

### Reagents and solutions

All reagents were purchased from Sigma Aldrich unless otherwise noted. Cell culture media was obtained from ThermoFisher Scientific and Lipidure®-CM from AMS Biotechnology (Europe) Ltd. The enzymes used for cell isolation were collagenase Type F, collagenase Type 3 (Worthington, NJ, USA), papain (Worthington) and hyaluronidase. Cell culture dishes with glass coverslip bases (Ibidi µ-Dish 35 mm, high) were purchased from Thistle Scientific (UK). Tali™ Apoptosis Kit (Annexin V Alexa Fluor™ 488) and Dil-conjugated oxLDL from Human Plasma (Dil-OxLDL) were both from Invitrogen™ (ThermoFisher Scientific). The antibodies used for immunocytochemistry were mouse anti-SMA-Cy3 (C6198, Sigma-Aldrich), rabbit anti-Galectin 3 (PA579595 ThermoFisher Scientific) and goat anti-rabbit-AlexaFluor633 (A21071, ThermoFisher Scientific), along with Hoechst33342 Solution (ThermoFisher Scientific).

The buffers used during cell isolation were: Mops buffer (145mM sodium chloride, 2mM MOPS, 4.7mM potassium chloride, 1.2 mM monosodium phosphate, 5mM glucose, 0.02 mM EDTA, 2mM sodium pyruvate, 1.2mM magnesium chloride, 2mM calcium chloride, pH 7.4) and isolation buffer (80mM sodium glutamate, 55mM sodium chloride, 6mM potassium chloride, 10mM glucose, 10mM Hepes,1mM magnesium chloride, 0.1mM calcium chloride, 0.2mM EDTA, pH 7.4), with or without 2 mg/ml fatty acid free bovine serum albumin (BSA).

### Device fabrication and functionalisation

Microwell array devices consisted of a functionalised, 100 μm thick (11 × 11 mm) through-hole membrane fabricated in PDMS (Sylgard 184, Dow Corning), with through-hole widths ranging from 100 to 140 μm, and bonded to a glass coverslip-bottomed culture dish. The through-hole PDMS membranes were fabricated by soft lithography, adapting a previously reported method (Hsu et al. 2004). A microfabricated mould was created by standard photolithographic patterning of SU-8 (SU-8 3035, Chestech Ltd) on a polished silicon wafer. PDMS, with a ratio of 10:1 w/w elastomer:curing agent was mixed and degassed before being poured over the mould, which had previously been silanised by vapour deposition of 1 H,1 H,2 H,2 H-perfluorooctyl-trichlorosilane to produce a mould release layer. An acetate sheet was placed on top of the uncured elastomer layer and two glass slides were placed on either side of the acetate-mould assembly to achieve a uniform distribution of pressure within a hydraulic hot-press (Specac, UK), which was set to 80°C, 0.2 tonne pressure for a minimum of 1 h. Following cooling of the assembly, the cured PDMS through-hole layer was peeled off from the master and cut to remove uneven edges.

Surface functionalisation of the PDMS microwell array was tested using two methods - by contact printing and by flood treatment - and utilising two cell-repellent compounds, Synperonic®-F108 and Lipidure®-CM. Flood treatment of the microwell arrays required the PDMS through-hole layers to be temporarily bonded by conformal contact to a sacrificial glass slide. Whilst attached to this slide, they were immersed in a solution containing the cell-repellent coating. Plasma treatment of the PDMS through-hole layer was performed prior to immersion in a 1% (w/v) Synperonic®-F108 solution, with an overnight incubation. Surface functionalisation with Lipidure®-CM did not require plasma treatment and was achieved by immersion in a 1% (w/v) Lipidure®-CM solution (in ethanol) for 1 min, before baking at 50°C for 1 h. Treated PDMS through-hole layers were carefully peeled from the sacrificial glass slide before being transferred to a glass coverslip-bottomed dishes. If air bubbles formed between the PDMS and glass layers, degassing of the device within a vacuum chamber was performed. Serum-free cell culture media was the added to the devices which was stored in the incubator until use (a maximum of 2 days).

Alternatively, contact printing was performed, using Synperonic®-F108 solution as the ink, onto a PDMS through-hole membrane that was permanently bonded to a glass coverslip-bottomed dish using oxygen plasma surface treatment (Pico plasma cleaner, Diener electronic, Germany). The bonded array was thermally cured for 30 min at 80°C and stored dry at room temperature until required. Prior to stamping the microwell arrays were plasma treated again. Flat PDMS stamps were created by casting onto a polished, silanised silicon wafer and then cutting to size (12 × 12 mm), before sterilising by immersion in 70% ethanol alongside the plasma bonded arrays. Following rinsing with sterile deionised water, stamps were inked with a 1% (w/v) Synperonic®-F108 solution for 30 min. Stamps were rinsed with sterile DI water and air dried for 5 min before being placed ink side down onto the upper surface of the array, gently tapping the stamps to ensure contact, and left for 15 min. Immediately after removal of the stamp, the microwell array devices were filled with serum-free SMC medium.

After the addition of media, all devices were placed inside a vacuum desiccator to remove any air bubbles that formed within individual microwells within the arrays during media addition. Devices were then placed inside an incubator at 37 °C and 5% CO_2_ overnight before use.

### Cell isolation

Native smooth muscle cells were freshly isolated from rat tissue as described in Sandison et al. (2016). Animals were not subject to any other treatments and killing was in accordance with UK regulations (Animals (Scientific Procedures) Act 1986. Aortic and carotid artery vessels were dissected from male Sprague Dawley rats (10–12 weeks old), euthanized by an intraperitoneal overdose of sodium pentobarbital. Tissues were immediately placed into Mops buffer after harvesting and surrounding connective tissue was removed by manual dissection. SMCs were then isolated from each tissue by a series of enzymatic digestions followed by trituration (with all incubations carried out in BSA-containing isolation buffer and in a water bath maintained at 35.5 °C, with all tissues washed with this buffer after each enzyme incubation). Tissues were first incubated in 2.0 mg/ml collagenase Type 3 (aorta, 40 min; carotid, 30 min) to aid subsequent removal of the adventitia using a pair of fine tweezers. Vessels were then cut open and denuded of endothelium, before an incubation (aorta, 30 min; carotid, 20 min) in collagenase type F enzyme (2.2 mg/ml) with hyaluronidase (1.0 mg/ml).The final digestion step was carried out in 1.7 mg/ml papain with 0.7 mg/ml dithioerythritol (aorta, 30 min; carotid, 20 min). To produce a suspension of single native SMCs for direct plating into microarrays, digested tissue sections were washed several times in a sterile solution of BSA-free isolation buffer within a cell culture hood. They were then triturated using a series of sterilised fire-polished glass pipettes with decreasing bore sizes, with a small volume of isolated cells from each trituration viewed under a microscope to determine the best trituration for use based upon cell morphology and the density of the cell suspension.

### Smooth muscle cell culture in microwell arrays

An aliquot of native, freshly isolated SMCs was pipetted into a microwell array device, seeding ~ 1 × 10^4^ cells across the array. These cells were initially imaged *in situ* within microwells in serum-free culture medium containing 1:1 Ham’s F-12: Waymouth’s media, supplemented with 1% L-glutamine, 1% penicillin-streptomycin and 1% amphotericin B. Following this, SMCs were exposed to 10% FBS to induce phenotypic modulation and were maintained in serum-containing culture media at 37 °C in 5% CO2 and 80% humidity thereafter. Media changes were carried out once every two days. For apoptosis staining at 24 and 72 h, an annexin-V Alexa Fluor 488 solution was added (concentrations as stated in the manufacturer’s protocol) to the microwell array devices and incubated for 20 min at room temperature in the dark before imaging. Where noted, at day 6, cells were exposed to Dil-OxLDL (10 mg/mL in serum-free culture media with 0.3% BSA) for 24 h. Following this, the cells within arrays were washed gently (5x) with phosphate buffered saline (PBS) prior to live-cell imaging and subsequent fixation.

### Acquisition and analysis of microscopy images

To track cell fate, the SMCs within the arrays were monitored every 24–48 h using either an inverted Observer A1 microscope (Zeiss) or an inverted Ti-U microscope (Nikon, UK), both connected to an Orca Flash 4.0 camera (Hamamatsu), for bright-field/phase contrast and fluorescence imaging using a 10x or 20x objective. Images were analysed and data processed using ZEN Blue (Zeiss), ImageJ (NIH) and Nikon Elements (Nikon) software. A flat-field image was obtained for each fluorescence channel (fluorescent microscope slides, Thorlabs, UK) and was used to correct epifluorescence images for uneven illumination prior to analysis of Dil-OxLDL and Galectin-3 staining. Individual cell regions of interest (ROIs) were manually drawn, zooming-in on individual microwells and using a combination of phase contrast and fluorescence images.

### Immunocytochemistry

Cells in the microwell arrays were fixed and stained on day 7 of culture. Cultures were first washed with PBS before fixing with formalin (10% neutral buffered formalin, 25 min) and quenching with 100 mM glycine (pH 7.4). Cells were then permeabilised with Triton X-100 (0.2%, 10 min) and rinsed with PBS before blocking with BSA (2% in PBS, 30 min). Cultures were incubated at room temperature for 1 h with either primary conjugated (anti-SMA-Cy3, 1:100 dilution) or unconjugated (anti-Galectin-3, 1:250 dilution) antibodies, followed by 1 h in secondary antibody for the latter (anti-rabbit-AlexaFluor633, 1:200 dilution), with Hoechst33324 (1:2000 dilution) added at the end of these incubations for 10 min in staining solution (2% BSA in PBS with 0.05% Tween-20). All stained samples were imaged using the same recording conditions and all images processed using the same operations as described below. Regions of interest (ROIs) were manually drawn for all individual cells in tracked microwells, based on day 7 images obtained during oxLDL imaging. If necessary (as a consequence of slight cell movement during fixation), ROIs were subsequently adjusted for the Galectin-3 stained images.

### Statistical analysis

Statistical analysis was carried out using GraphPad Prism 8.1.2 (GraphPad Software Inc., San Diego, CA). All values are quoted as mean ± standard error of the mean (SEM) unless otherwise stated. Results with a *p* value of < 0.05 were considered to be significant and full details of the statistical tests used are given in the relevant figure legends and associated text.

## Results and discussion

### Microdevice architecture

**Fig. 1:**
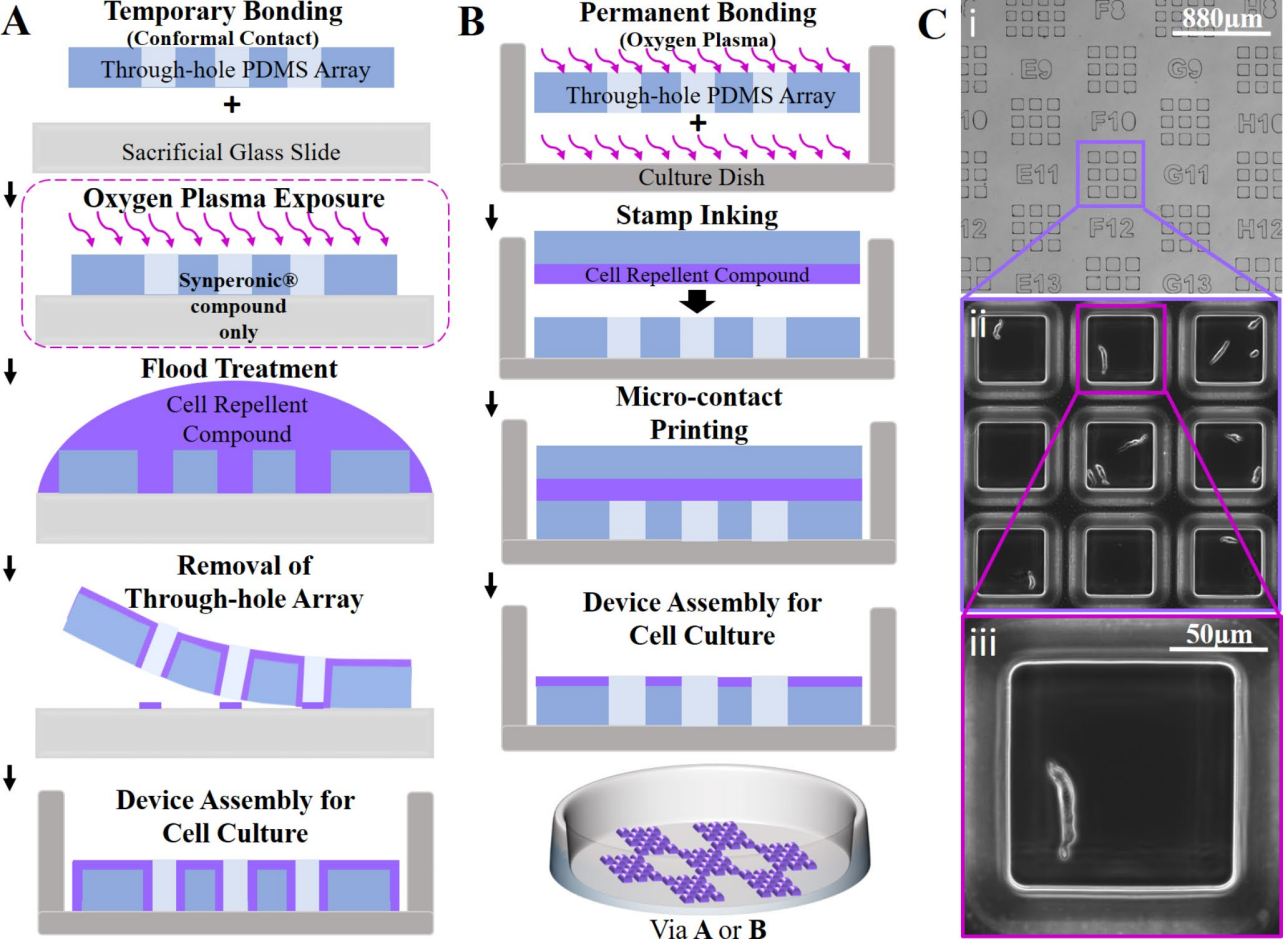
*Microwell device fabrication and architecture.* Schematics of the two surface functionalisation methods used to achieve hybrid cell-repellent/cell-adhesive regions within the microwell array. (A) Flood treatment approach, utilising conformal bonding, that achieved complete coverage of the cell-repellent compound across all exposed PDMS surfaces. (B) The use of a contact printing approach that results in a cell-repellent coating only on the top surface of the PDMS through-hole membrane. (C) Microscopy images showing a number of grids with their address labels (i) that were subsequently seeded with native carotid artery SMCs (ii), with SMCs displaying their characteristic elongated morphology (iii, a native SMC within a 120 μm well)

To study the individual fate of single vSMCs, functionalised PDMS through-hole membranes were used to create a device with over 2000 independent square microwells (Fig. 1), grouped together into grids of microwells with equal dimensions with 98 addressable grids per device. The overall dimension of each grid matched the field of view when using a 20x objective. Each microwell guaranteed confinement of the cells captured within and, with our seeding conditions and freshly isolated cells, resulted in the tracking of typically ~ 150 single, primary cells over the course of 1 week. The lower number of tracked cells with respect to the total number of microwells stems from a combination of the distribution of the number of cells per well across the array along with the high level of apoptosis typical for these primary cells, as discussed below. Membrane thickness, defining the microwell depth, was measured using an Alpha-Step profilometer to be approximately 100 μm, a value adequate for confining non-adhered cells to the microwells during early washing steps (a critical time point as these primary cells typically required 24–48 h to fully adhere).

### Surface functionalisation approaches: flood treatment & contact printing methods

To ensure reliable single-cell tracking of adherent, migratory cells over several days of culture through steadfast confinement to individual microwells, surface functionalisation of the PDMS through-hole membrane with a cell-repellent coating was essential, whilst leaving the glass base of each microwell unmodified. Despite its innate hydrophobicity, native PDMS alone did not prevent cells from migrating out of the microwells (SI Fig. 1A). Therefore, to achieve cell confinement, two functionalization methods and two cell-repellent compounds were assessed to create a robust cell-repellent coating on the PDMS membrane: a flood treatment technique (Fig. 1A), where the top PDMS surface and the microwell walls were functionalised, and a microcontact printing technique (Fig. 1B), where the top PDMS surface only was functionalised. Coatings were considered to be effective if complete inhibition of cell adhesion was maintained over a 7 day long assay.

The first method (Fig. 1A) involved flood treatment of the through-hole PDMS membrane whilst it was conformally bonded onto a temporary glass support, so that the membrane could be easily removed after surface functionalisation and so that its bottom surface remained uncoated to ensure the success of a second conformal bonding step. Here, Synperonic®-F108 (a PEG-based surfactant successfully utilised in microfluidic devices to achieve ultra-low adhesion condition for the formation of cancer spheroids (Mulholland et al. 2018)) and Lipidure®-CM (a biocompatible, synthetic polymer, which is highly resistant to cell and protein adsorption through the action of the repeating phosphoryl choline unit; Kaneko et al., 2020), were tested as cell-repellent compounds. The entire membrane-glass assembly was submerged in a solution containing one of the cell-repellent compounds to produce a protein resistant coating over the entire exposed surfaces (e.g. side-walls and top surface) of the membrane. In the case of Synperonic, this flood treatment was carried out after oxygen plasma treatment to facilitate adsorption of the triblock copolymer (Tan et al. 2004). The functionalised PDMS membrane was then peeled off and carefully placed on a glass coverslip-bottomed dish, taking care to avoid air bubble entrapment between the PDMS and glass. This conformal bonding method did not result in any membrane detachment from the substrate for the duration of the assay.

When using Synperonic®-F108, addition of cells to flood treated arrays, showed effective confinement of dividing cells from day 0–5. However, on many occasions, progeny from highly proliferative cells did not stay confined beyond 5 days (60% of devices exhibited some failure of confinement, n = 15), with cells migrating out of the wells onto the top surface of the PDMS (SI Fig. 1B). This effect could be due to adsorption of proteins from the cell culture media over time (Li et al. 2015) or possibly due to damage of the cell-repellent coating during transfer of the through-hole membrane from the glass slide to dish (e.g. a consequence of flexing). However, when using Lipidure®-CM, where surface modification and device assembly was completed in less than 2 h, effective cell confinement was achieved for the 7-day assay period and beyond: indeed, when cells were cultured for > 3 weeks in a Lipidure-coated device, the cell-repellent coating did not fail and cell confinement was maintained (see SI Fig. 1C). The improved confinement of Lipidure-coated devices (across 49 experiments with Lipidure coated devices, no failures occurred) could potentially have been due to more actively proliferating cells within the Synperonic® devices. However, after analysis of cell proliferative capacity (see below), similar levels of proliferative capacity were seen across arrays for both types of cell-repellent compounds.

The second method (Fig. 1B) consisted of using contact printing to stamp Synperonic®-F108 onto the upper surface of the through-hole PDMS membrane. Contact printed arrays required less handling of the thin through-hole membrane and allowed for plasma-bonding of the PDMS to the glass. However, the main drawback of this approach was that, unlike flood-treated membranes, SMCs were able to anchor themselves to the inner sidewalls of the microwell and suspended themselves from one wall to another (SI Fig. 1D). This resulted in different microwells requiring imaging at different focal planes, hindering fast acquisition of images. In addition, as with the Synperonic flood-treated device, cells present in highly proliferative microwells were able to overcome the cell-repellent coating and migrate across the top surface of the membrane (67% failure rate, n = 6).

The performance of the Lipidure-coated, flood-treated devices was superior to the others and constituted the most promising method for the long-term confinement of single, adherent cells and for the imaging of their fate in a medium-throughput fashion. It was therefore selected for all the experiments reported below. The majority of single-cell array studies have been carried out over a short period of time (e.g. ≤4 days) (Dykstra et al. 2006; Yamahira et al. 2014; Rodriguez-Moncayo et al. 2020; Zaretsky et al. 2012; Zhang et al. 2020; Nowotarski et al., 2020), which limits the extent of data collection. Reported examples of longer culture periods (e.g. 7 days; Chang et al., 2015) have used devices with microwell surfaces that, as demonstrated here, would not contain highly proliferative vSMCs. By extending the culture period through the use of Lipidure-coating of the through-hole membrane, a greater understanding of the extent of heterogeneity in phenotypic change and in the proliferative capacity of single vSMCs *in vitro* becomes possible.

### Single vSMC tracking within microwell arrays

**Fig. 2:**
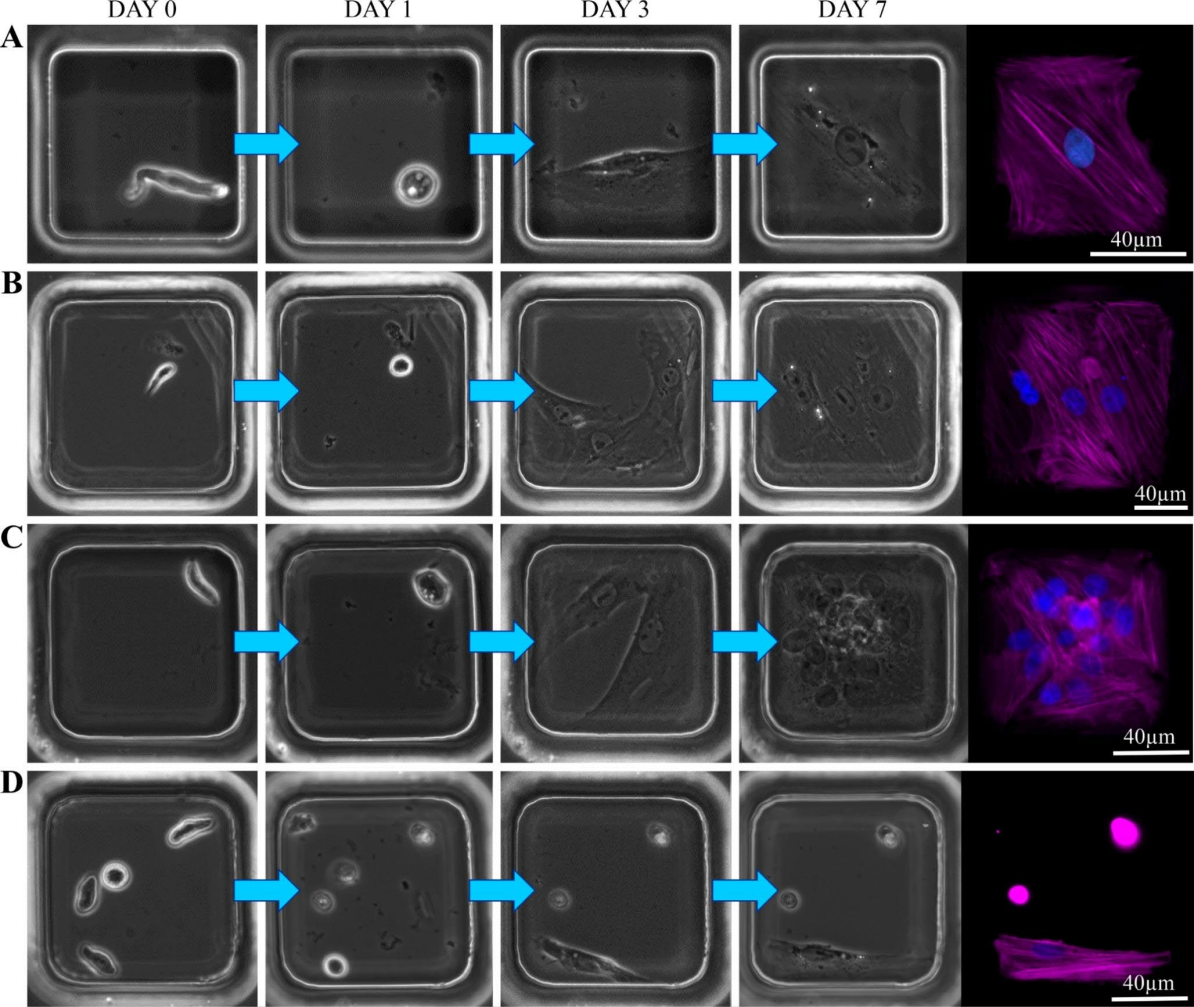
*Example time-courses showing vSMC tracking in microwell array devices*. Phase contrast images show changes in cell morphology and proliferation over 1 week, with endpoint ICC staining on day 7 (magenta for SMA; blue for Hoechst) to facilitate cell counting. Four different example scenarios are shown, with the native cells displaying their characteristic elongated morphology: (**A**) a single starting cell remaining as a single, non-dividing cell throughout; (**B**) a single starting cell which proliferates to produce a 3-cell well by day 7 (nuclei count of 4 stems from a multinucleated cell, with the 2 nuclei abutting each other); (**C**) a single starting cell which divides rapidly after day 5, resulting in a confluent well (containing 12 cells) by day 7; and (**D**) a microwell with 4 starting cells which, through apoptosis, becomes a single-cell well by day 2

To demonstrate the ability to perform single cell tracking of vSMC phenotypic modulation, thoracic aorta and carotid artery vSMCs were freshly isolated from rat tissue. As previously reported (Sandison et al. 2016), the cell suspensions resulting from the multi-stage enzymatic digestion employed contained pure populations of native vSMCs, which displayed their characteristic elongated morphology (Fig. 1Ciii). As serum induces phenotypic modulation in vSMCs (with cells rapidly rounding up in response to serum exposure before subsequently spreading outwards onto the substrate, adopting a radically different morphology), cells were seeded into the prepared microwell arrays in serum-free media to enable initial imaging of the native vSMCs (Day 0 in Fig. 2). Following the introduction of serum to stimulate the transition to a proliferative, migratory phenotype, the microwell arrays were imaged at 3–4 time-points over the course of 1 week (e.g. on days 2, 5 and 7, as in Fig. 2). vSMC confinement to a specific addressable microwell ensured that the same cell could be readily tracked at fixed time-points alone, negating the need for continuous monitoring by time-lapse microscopy and allowing for the monitoring of multiple cultures areas across a device, thus substantially increasing throughput.

To be an efficient approach for single-cell tracking, microwell design and cell-seeding strategies must generally be optimised to achieve a high rate of single-cell occupancy. However, many primary cell types experience high levels of apoptosis when initially cultured (Hu et al. 2015; Vinken et al. 2014; Kaviani et al. 2019), including native smooth muscle (Sandison et al. 2016). Therefore, seeding with a cell density that resulted in higher initial cell numbers (e.g. targeting a distribution of primarily 1–3 cells per well) was a more effective strategy for increasing tracking throughput, as many microwells initially containing 2–3 cells gave rise to a single viable cell (Fig. 2D) by 48-72 h (the vast majority of apoptosis having occurred by this time). Employing a live-cell fluorescence apoptosis assay (Annexin V detection of phosphatidylserine residues) enabled the reliable identification of wells with a single viable cell for subsequent tracking (SI Fig. 2), and this approach was used in the results presented below. Our seeding protocol was optimised to account for inherent variations between fresh cell isolations and to minimise the time between tissue trituration and the imaging of native cells within the microwell array, as, even within serum-free media, vSMCs will contract and round-up with time.

Following the introduction of serum, vSMCs rounded up fully before adhering to and spreading outwards over the base of the microwells (this occurred at varying rates for different cells over the first 3 days, as reported previously [2016]), after which the onset of proliferation could occur. When tracking vSMCs over a 7 day period (the point at which the progeny of the most proliferative cells had packed the microwell surface to confluency) considerable diversity in proliferative capacity was observed (Fig. 2). It should also be noted that SMCs can on occasion attempt to divide but do not subsequently complete the process (e.g. they undergo mitosis but not cytokinesis, as illustrated in SI Fig. 3); this results in a single multinucleate cell (Fig. 2B).

### Quantification of heterogeneity in single vSMC proliferative capacity

**Fig. 3:**
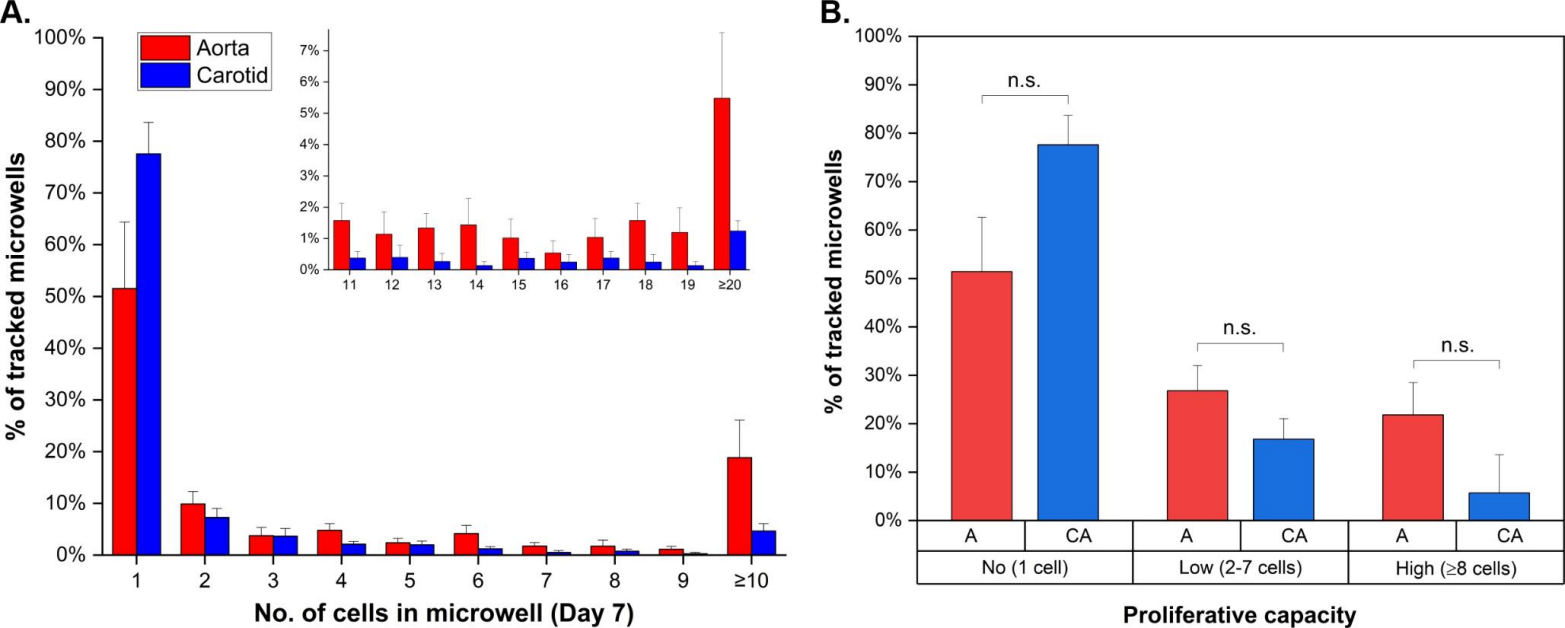
*Heterogeneity in the proliferative capacity of vSMCs.* Results shown for tracked single aorta and carotid vSMCs after 7 days *in vitro* (with oxLDL introduced on day 6 here). Plots show both the broad range of cell progeny numbers possible (**A**), inset highlighting clonal populations with ≥ 11 cells) and the classification of cells into no-, low- and high-proliferator groups (**B**). 2-sample t-tests were used to compare aorta (A) and carotid artery (CA) populations (p < 0.05 considered significant; *n* = 3 animals)

To quantify the extent of heterogeneity in the capacity of individual vSMCs to proliferate, the number of cells present in each microwell (e.g. the progeny of the original parent cell) was counted at day 7 and the total number of microwells with a given cell count calculated, expressing this as a percentage of the total number of microwells containing trackable single cells following apoptosis staining. Two different vascular tissue types were investigated: aorta (A) and carotid artery (CA) (Fig. 3). The majority of cells remained as single, non-dividing cells (n = 3 animals; 51% non-proliferators A, 78% CA), with a lower number of cells undergoing a single round of cell division (10% A; 7% CA). There was, however, a small sub-population of cells that showed an extremely high proliferative capacity (inset in Fig. 3A; with 18% & 5% of A & CA cells producing ≥ 10 progeny). This finding is in-line with the observation from a range of other studies (Espinosa-Diez et al. 2021) that a limited number of medial SMCs selectively expand to populate atherosclerotic lesions, whilst providing direct evidence that a substantial sub-population of SMCs exhibit at least some capacity for proliferation and that there is considerable diversity in the extent of the resulting clonal populations.

Because of the range in the resulting number of progeny, we classified (Fig. 3B) each tracked cell as either a non-proliferator, low proliferator (2–7 cells in microwell on day 7) or high proliferator (≥ 8 cells), with the latter corresponding to reaching at least the 3rd generation of daughter cells (generation reached being dependent upon how many daughter cells go on to divide). This facilitates straightforward comparisons between different experimental conditions or cell populations. For example, related to points raised above, quantification showed no difference in proliferation levels when comparing Synperonic and Lipidure-coated devices (SI Fig. 4). When comparing tracked microwells that contained apoptotic cells (e.g. started out with more than one cell) and those that did not (e.g. started out as a single cell), for aortic vSMCs (SI Fig. 5A) there was no difference in percentage of cells demonstrating no, low or high proliferative capacities. For carotid artery cells (SI Fig. 5B), there was a significant difference in the percentage of cells that were non-proliferative or demonstrated a low level or proliferation. However, there was no significant difference in the percentage of cells classified as high proliferators. Furthermore, when comparing all tracked vSMCs from the two vascular tissue types investigated (Fig. 3B), although the number of proliferative cells was larger for aortic populations (for 49% of cells there was at least one proliferation event) than for carotid artery (22%), there was no significant difference between them for any category (p = 0.166/0.215/0.149 for no/low/high). Therefore, subsequent analyses were performed on a single tissue type (carotid artery) only.

By quantifying proliferative capacity at the single-cell level, these microarray device are amenable to *in vitro* screening of potential therapies that target highly proliferative vSMC drivers of atherosclerosis. They also provide a new tool with which to develop a better understanding of other, potentially diverse, characteristics of proliferative vSMCs and to investigate whether further types of sub-population are present.

### Single-cell tracking of vSMC oxLDL uptake and macrophage marker expression

A range of *in vitro* and *in vivo* studies have provided substantial evidence that vSMCs can adapt their phenotype to acquire functions of plaque foam cells that were previously attributed to macrophages (Rong et al. 2003; Feil et al. 2014; Sandison et al. 2016; Wang et al. 2019; Jacobsen et al. 2017), including phagocytosis and lipid uptake. oxLDL is a key driver of atherosclerosis acting through several mechanisms including through the promotion of foam cell formation. Therefore, we investigated whether heterogeneity in oxLDL uptake was observed within tracked populations of vSMCs and, if so, whether this heterogeneity was correlated to proliferative capacity, simultaneously staining all tracked cells for the macrophage marker Galectin-3, previously shown to be upregulated in both *in vivo* and *in vitro *models of phenotypically modulated vSMCs (Rong et al. 2003; Alencar et al. 2020).

**Fig. 4:**
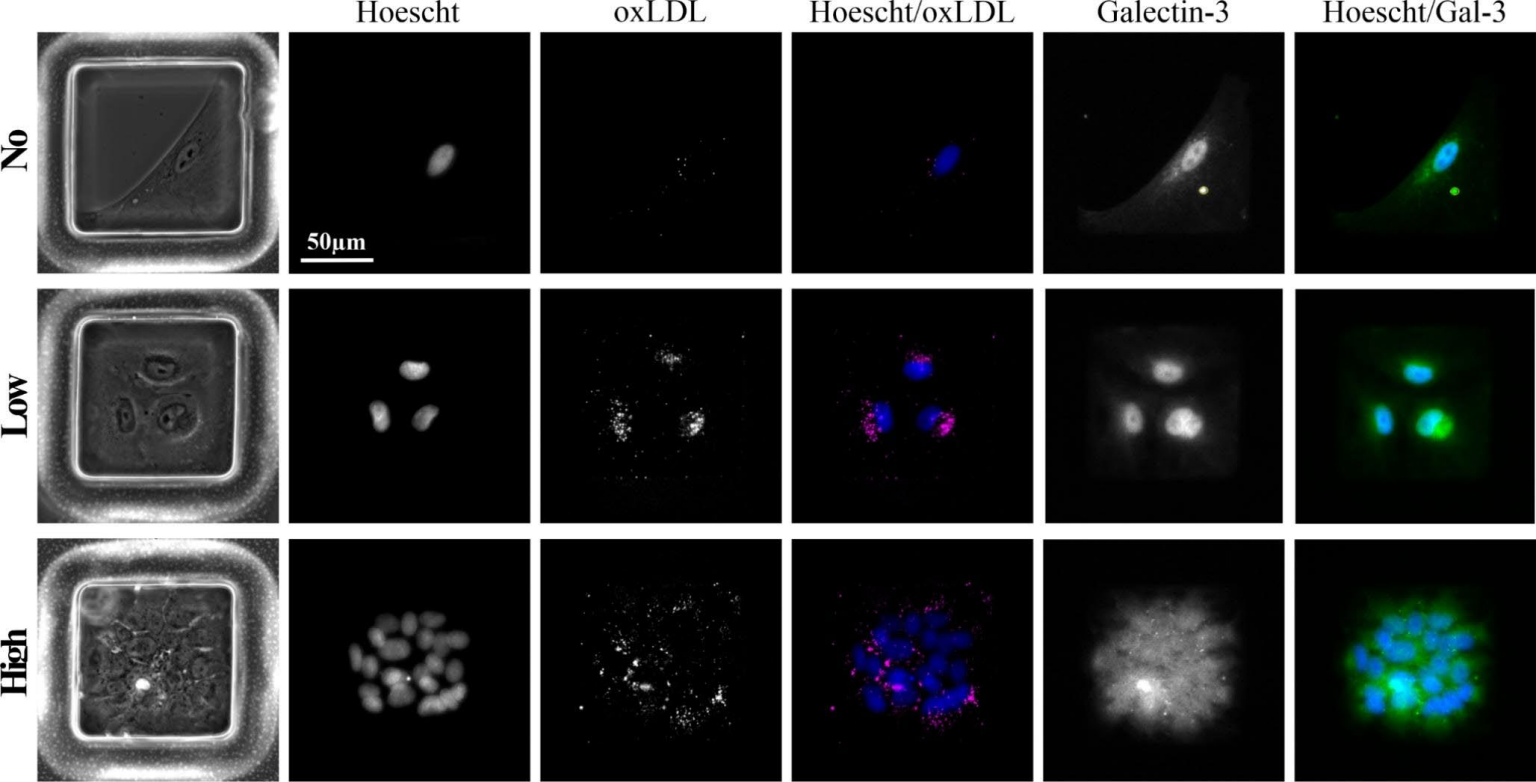
*Single-cell tracking of oxLDL uptake and Galectin-3 expression in carotid artery vSMCs*. Shows examples of uptake of Dil-oxLDL (day 7, live cells) and expression of Galectin-3 (day 7, after fixation) for microwells containing the progeny of native SMCs with “no” (phenotypically altered parent cell in this case), “low” or “high” proliferative capacity. Representative images illustrate the overall increase in mean oxLDL uptake (magenta) observed for proliferating cells, along with higher intensity Galectin-3 staining (green). Nuclei were counterstained with Hoescht (blue)

Therefore, an oxidised low-density lipoprotein live-cell imaging assay and quantification of Galectin-3 expression by immunofluorescence were performed. Dil-conjugated oxLDL (Dil-oxLDL) was added on day 6 to microwell array devices seeded with carotid artery vSMCs and incubated for 24 h prior to live-cell imaging, following which the culture was fixed and cells stained for galectin-3 (the antibody used validated by positive staining of a bone-marrow derived murine macrophage cells and by negative staining of fixed freshly isolated vSMCS, data not shown). As such, using our microwell array devices to track individual vSMCs, it was possible to assess whether there was any correlation between vSMC proliferative capacity and oxLDL uptake or Galectin-3 expression at the single-cell level. Heterogeneity in the uptake of ox-LDL was observed across the array, with oxLDL being observed as distinct punctate staining within the cytosol and typically localised around the nuclei (Fig. 4). Galectin-3 staining was observed both within the cytoplasm and, more strongly, within the nucleus. Depending on the cell type and specific experimental conditions both subcellular locations have been reported previously (Haudek et al. 2010), including a similar distribution between the two in cultured SMCs (Gaudin et al., 2012).

**Fig. 5:**
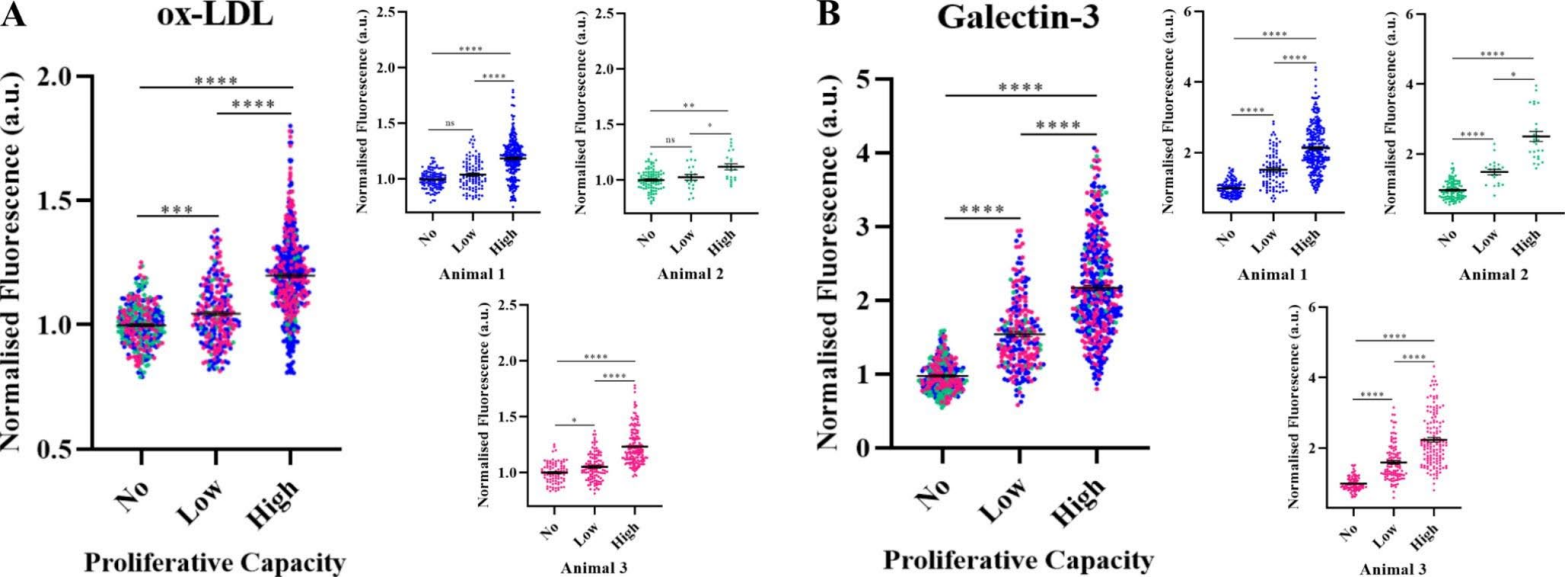
*Increased uptake of oxLDL and Galectin-3 expression with proliferative capacity*. Mean normalised whole-cell fluorescence intensity measurements from Dil-oxLDL uptake (A) and Galectin-3 staining (B) of tracked single carotid artery vSMCs, segmenting all individual cells within a microwell and normalizing each data point to the mean value for the “no” proliferation category (after first performing flat-field and background corrections). Significance levels resulting from Kruskal-Wallis with Dunn’s multiple comparisons tests are indicated as: ns, p > 0.05; *, p < 0.05; ***, p < 0.001; ****, p < 0.0001

Fluorescent signals from Dil-oxLDL and Galectin-3 staining were quantified by measuring the flat-field and background corrected mean for each individual cell (creating single cell ROIs for each) for carotid artery vSMCs from 3 animals. The normalised mean fluorescence (normalising each data point to the mean value for the “no” proliferation category) was then plotted against proliferative capacity (Fig. 5), with each data point displaying the value from an individual day 7 cell. The results from both individual experiments (that is tracking the cells from a single animal) and for all experiments combined show a statistically significant trend of both increasing Dil-oxLDL uptake and increasing Galectin-3 expression with increased proliferative capacity. This confirms the presence of highly proliferative vSMC sub-populations, which result in Galectin-3 positive clonal populations with an increased ability to uptake oxLDL, that thus have the potential to develop into SMC-derived foam cells.

Many different characteristics have been attributed to phenotypically altered vSMCs, however diversity in the extent of their expression within vSMC populations remains largely unclear. *In vitro* single cell fate mapping approaches provide one route to better understanding this and our microwell array approach, with its effective long-term cell confinement, enables simultaneous characterisation of multiple characteristics (including functional assays through live-cell imaging) and thus classification of individual vSMCs into different sub-populations. Whilst *in vitro *models are necessarily simplified systems that do not maintain the complexity of the *in vivo* environment, they do permit targeted questions to be more readily asked. For example, as recently noted by Espinosa-Diez et al. (2021), advanced *in vivo *clonality tracking methods (based on the inducible and conditional expression of reporters in transgenic animals) can enable the identification of cells that derive from a common precursor but they cannot determine whether these precursors are the only medial SMCs that retain the ability to proliferate. Our approach described here addresses this, enabling quantification of the extent that individual medial SMCs can proliferate under given experimental conditions, whilst simultaneously analysing functional characteristics (e.g. oxLDL uptake) of the resulting clonal populations.

## Conclusion

In this paper, we have demonstrated a novel methodology for the *in vitro* tracking of the phenotypic modulation of a large number of single, freshly isolated vSMCs using functionalised microwell arrays. To ensure robust, long-term cell confinement, we tested different surface functionalisation approaches for creating hybrid cell-repellent/cell-adherent microstructures, identifying Lipidure-CM as a highly effective compound for creating long-term cell-repellent surfaces, and have validated protocols for monitoring these cultures, using a combination of microscopy-based assays. A distinctive advantage of this approach is the parallel nature of the assays, mapping hundreds of individual microwells over several experimental time-points, whilst enabling long-term culture (> 3 weeks) and confinement of adherent cell types. The system enables the detailed interrogation of single cells, assessing distinct phenotypic characteristics to determine population variability across different tissue-specific traits, with the potential for identifying previously unrecognised cell sub-populations and, with extended fixed time-point imaging, for contributing to our understanding of the dynamic processes implicated in cell plasticity. We have employed our microarray device to quantify the diversity in the proliferative capacity of vSMCs at the single-cell level, quantifying the presence of a highly proliferative vSMC sub-population and demonstrating that proliferative clonal populations were associated with higher levels of oxLDL uptake and of Galectin-3 expression, in-line with reports that a limited number of phenotypically altered medial vSMCs populate atherosclerotic lesions. Moving forward, the microwell array approach could be readily employed with both vSMCs derived from human tissue and from transgenic animals, for example to correlate marker expression in native cells with ultimate proliferative capacity.

## Electronic supplementary material

Below is the link to the electronic supplementary material.

Supplementary Material 1
