# Supplementary material for: Cellular microarrays for assessing single-cell phenotypic changes in vascular cell populations
Source: Biomed Microdevices. 2023 Mar 16;25(2):11. doi: 10.1007/s10544-023-00651-5 (PMC10020314; doi:10.1007/s10544-023-00651-5)
Supplement: Supplementary file 1 — Supplementary Material 1 [file 10544_2023_651_MOESM1_ESM.pdf]

Cellular microarrays for assessing single-cell phenotypic changes in vascular cell populations

E. Smith [a,b], M. Zagnoni [a], M.E. Sandison *[b]

[a] Electronic & Electrical Engineering, Royal College Building, University of Strathclyde, Glasgow, G1 1XW, UK

[b] Biomedical Engineering, Wolfson Centre, University of Strathclyde, Glasgow, G4 0NW, UK

* corresponding author: mairi.sandison@strath.ac.uk

ORCiD (Smith): 0000-0002-5849-5785

ORCiD (Zagnoni): 0000-0003-3198-9491

ORCiD (Sandison): 0000-0003-1021-1461

Supplementary Information:

- SI Figure 1: Effect on cell confinement of untreated and functionalised PDMS surfaces
- SI Figure 2: Example time-courses illustrating the use of a live-cell apoptosis assay
- SI Figure 3: An example time-course of a failed cell division
- SI Figure 4: A comparison of the proliferative capacity in microwell arrays with different surface coatings
- SI Figure 5: The effect of apoptotic cells within individual microwells on vSMC proliferative capacity

SI Figure 1:

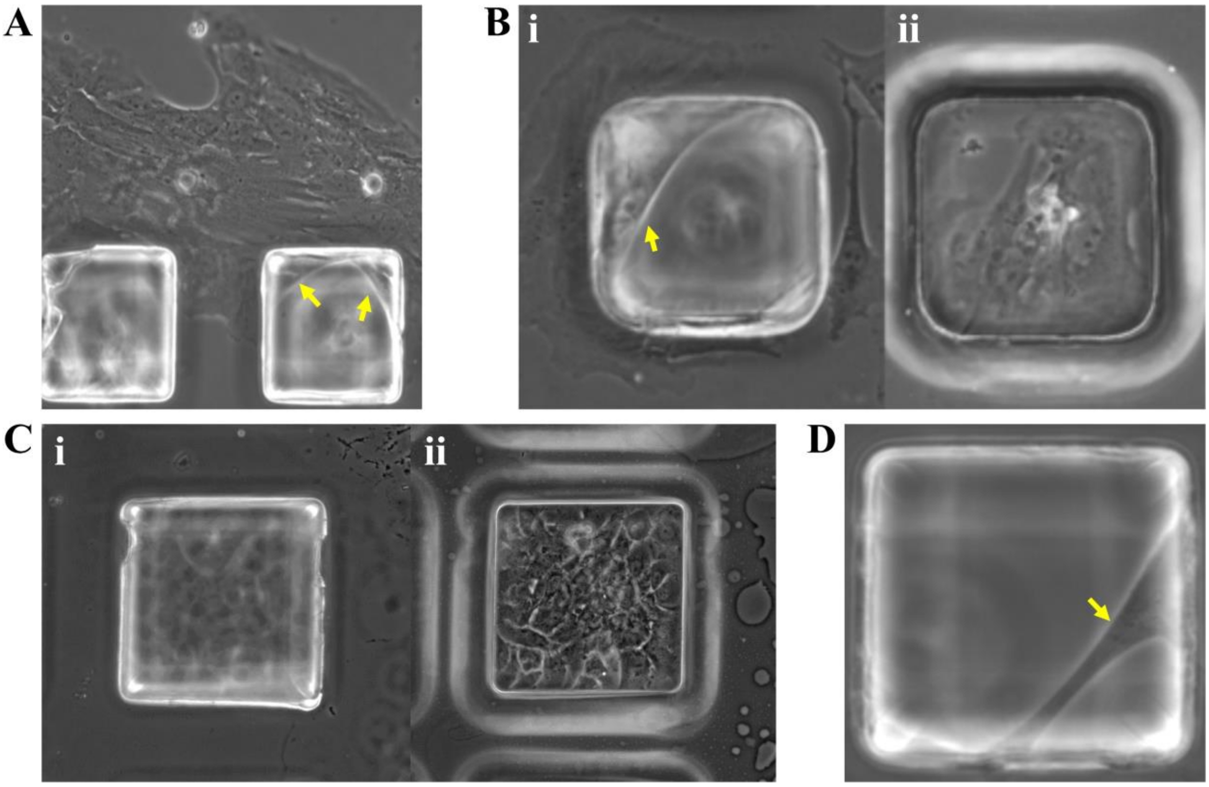

SI Figure 1. *Effect on cell confinement of untreated and functionalised PDMS surfaces*. (**A**) Substantial cell migration out of microwells onto the upper PDMS surface could be observed on devices where the PDMS through-hole membrane remained untreated. Example day 7 image shown, focussing on the upper surface of the PDMS membrane, arrows indicating vSMCs anchored to the sidewalls and appearing suspended over the microwell. (**B**) Example of vSMCs overcoming Synperonic®-F108 flood treatment, both migrating out of the microwell and anchoring themselves to the sidewalls (yellow arrows). Images (day 7) focussed on the upper surface of membrane (*i*) and focused on the base of the well (*ii*) are shown. (**C**) An example of extended vSMC confinement within Lipidure®-coated devices, showing a microwell after 25 days of culture with no cell escape from the microwell (*i*, upper surface) and a highly confluent cell culture at the base of the well (*ii*, culture surface). (**D**) A typical example of a vSMC suspended across an microwell, anchored onto an adherent side-wall in Synperonic®-F108 contact-printed device.

SI Figure 2:

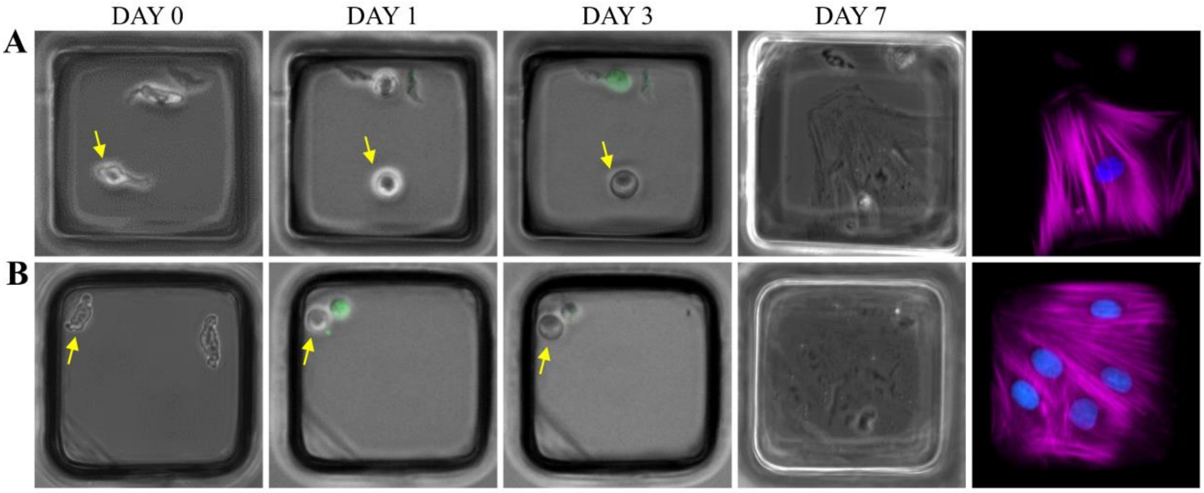

SI Figure 2. *Example time-courses illustrating the use of a live-cell apoptosis assay*. Phase contrast imaging in combination with fluorescence apoptosis staining at 24 and 72hrs enables identification of trackable single-cell wells for subsequent analysis. In both examples (**A**, an example where the surviving cell is non-proliferative; **B**, an example where the surviving cell does proliferate), the surviving the cell is indicated by a yellow arrow and the fluorescence signal from the Annexin V apoptosis assay is overlaid in green (Day 1 and Day 3). Day 7 staining was for SMA (magenta) and Hoechst (blue).

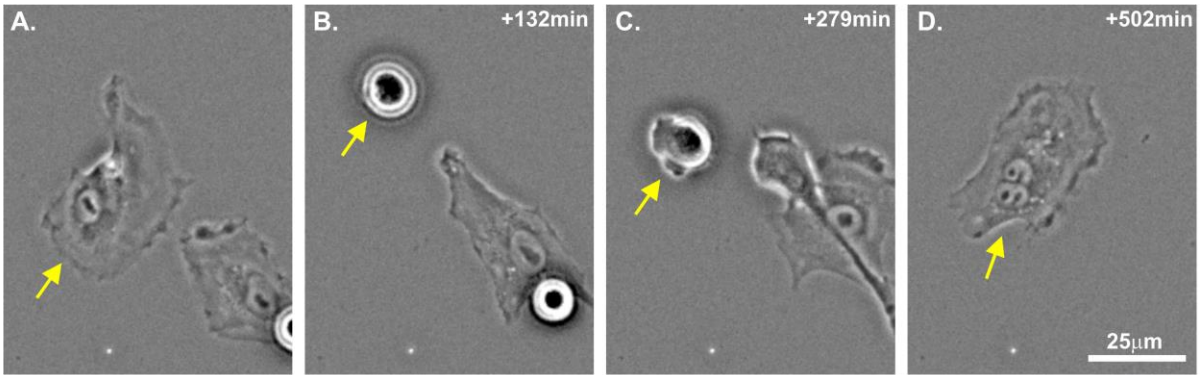

SI Figure 3. *An example time-course of a failed cell division.* In this sequence of images (taken from a time-lapse recording, with the culture maintained in a stage-top incubator), a carotid artery vSMC (tracked cell indicated by yellow arrow) is shown rounding up, as if dividing, but subsequently spreading outwards as a single cell with two nuclei (e.g. mitosis has occurred but not cytokinesis). The times indicated in the top right are the time elapsed since image *A*.

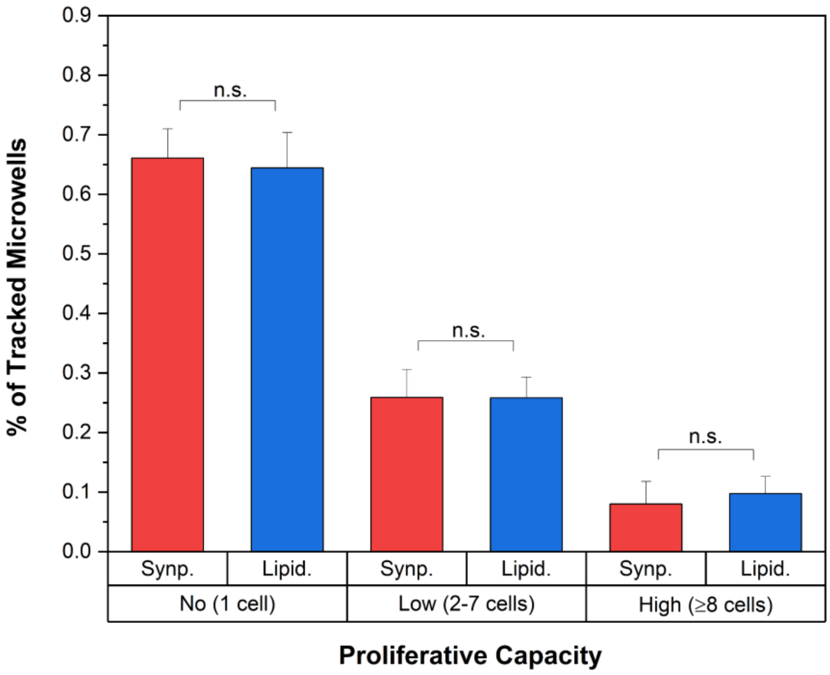

SI Figure 4. *A comparison of the proliferative capacity in microwell arrays with different surface coatings.* When comparing the proliferative capacity of tracked vSMCs (aorta, day 7 counts) in devices coated with either Synperonic®-F108 (Synp.) or Lipidure®-CM (Lipid.), no significant difference in the distribution of No, Low and High proliferators was observed (p=>0.999/>0.999/0.834 for no/low/high, Mann-Whitney test, n=5 devices).

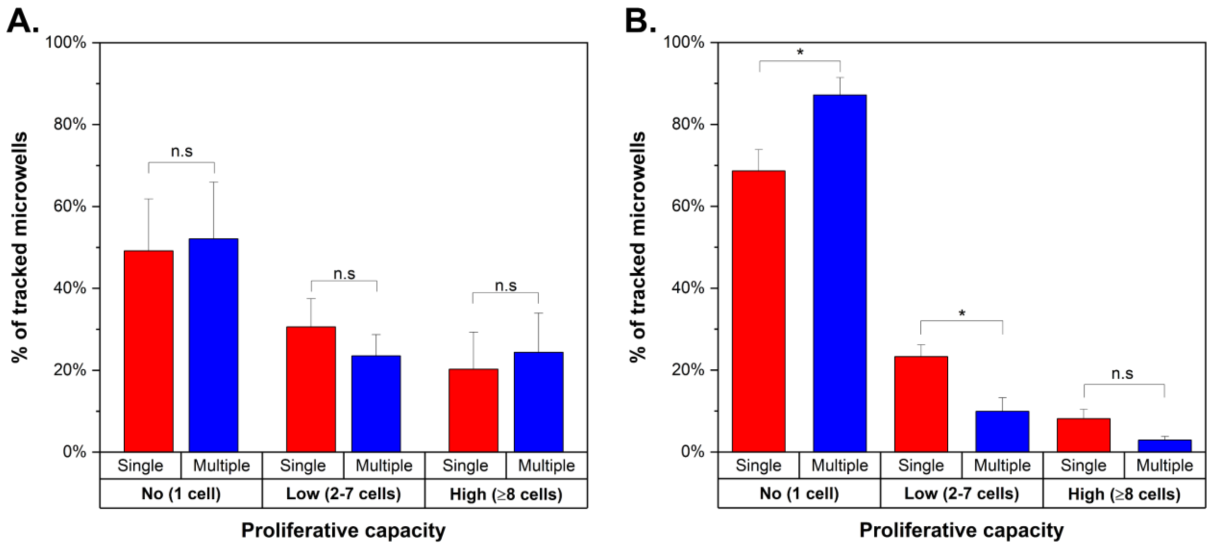

SI Figure 5. *The effect of apoptotic cells within individual microwells on vSMC proliferative capacity*. Results show the distribution in proliferative capacity of single vSMCs (day 7, no exposure to oxLDL) when the tracked cells were grouped into those that were the only cell seeded into their microwell ("single") and those that initially had more than one cell but became a single-cell well due to apoptosis of other cell/cells in the microwell ("multiple"). Data for aortic vSMCs (**A**) shows no significant difference between the two groups for any proliferative capacity category (p=0.881/0.446/0.765 for no/low/high, 2-sample t-test, n=4 animals). For carotid artery vSMCs (**B**), a slight shift in distribution was observed, though this was not significant for the high proliferator category (p=0.050/0.040/0.101, 2-sample t-test, n=3 animals).
